# An infant with patau syndrome associated with congenital heart defects

**DOI:** 10.1016/j.amsu.2022.104100

**Published:** 2022-07-02

**Authors:** Ubaid Khan, Ahmad Hussain, Muhammad Usman, Zain ul Abiddin

**Affiliations:** Department of Medicine, King Edward Medical University Lahore, Pakistan

**Keywords:** Patau syndrome, Trisomy 13, Patent ductal arteriosus, Atrial septal defect

## Abstract

**Introduction:**

and background: Patau syndrome or trisomy 13 is a clinically severe condition; 85 percent of patients die before reaching the age of one year, and the majority of children die before reaching the age of six months.

**Case presentation:**

This report discusses a case of a male infant, two days old diagnosed with Patau syndrome. After birth, his APGAR score was satisfactory. The initial clinical examination revealed cleft palate, cleft lip, and congenital clubfoot. A pansystolic murmur was heard at the left sternal border. The patient was managed according and was referred to a surgeon for pulmonary binding, PDA ligation, VSD closure, and repair of ASA with disbanding of the pulmonary artery.

**Clinical discussion:**

Studies have reported that patients with Patau syndrome present with cleft lip and palate, congenital heart defects, omphalocele, and holoprosencephaly. we also discovered dysmorphic characteristics such as the cleft palate and cleft lip, as well as serious congenital cardiac abnormalities. In addition, up to 80% of patients have been documented to have cardiac abnormalities, with patent ductus arteriosus, atrial septal defect, and ventricular septal defect.

**Conclusion:**

Patau syndrome is the third most common trisomy found in infants. The clinical manifestation of Patau syndrome includes cleft palate, cleft lip, limb impairments, and congenital heart problems. Despite the fact that early diagnosis and management prognosis is poor for patients suffering from Patau syndrome. Genetic counseling may be beneficial not just for increasing awareness of the diagnosis and its implications.

## Highlights

• This report discusses a case of a male infant, two days old, the third child of a non-consanguineous couple diagnosed with Patau syndrome.
• Patau syndrome is the third most common trisomy found in infants. The clinical manifestation of Patau syndrome includes cleft palate, cleft lip, limb impairments, and congenital heart problems.
• Genetic counseling may be beneficial not just for increasing awareness of the diagnosis and its implications but also for assisting families in making tough decisions about future care and family planning.

## 1 Introduction

**Fig. 1:**
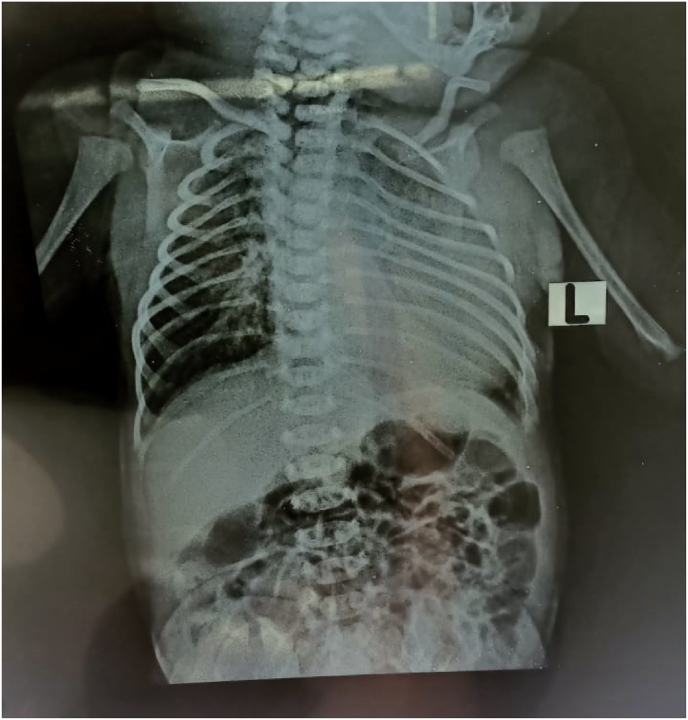
A section of the chest radiograph, X-ray findings revealed ….

Patau syndrome, a chromosomal abnormality, in which some or all of the cells of the body contain extra genetic material from chromosome 13. The extra genetic material disrupts normal development, causing multiple and complex organ defects. Thomas Bartholin discovered trisomy 13 in 1657, but it was Dr. Klaus Patau and Dr. Eeva Therman who discovered the chromosomal nature of the disease in 1960. Patau's sickness is named after him [1]. Patau syndrome, also known as trisomy 13, is the third most prevalent autosomal trisomy, with a live birth prevalence ranging from 1:7,000 to 1:29,000 based on the source of data. The patient cousin was diagnosed with a congenital syndrome so called “Omenn Syndrome” [2]. The average duration of survival is about 9 months, and 90% of patients die before they reach the age of one year [3] (see Fig. 1)

Normal development necessitates the presence of two (and only two) copies of the majority of the human autosomal genome; the occurrence of a third copy of an autosome is usually fatal to the growing embryo. As a result, trisomy 13 is unique in that it is one of only three autosomal trisomy's that can lead to live delivery. In fact, trisomy 13 is the most severe autosomal imbalance that can be endured by an embryo while still allowing it to live to term. Complex physiologic structures, such as those present in the CNS and heart, appear to be particularly vulnerable to chromosomal imbalance, either as a result of individual gene actions or as a result of the instability of developmental processes involving many genes operating simultaneously [4].

Microcephaly, midline facial deformities such as cyclopia, cleft lip and palate, microphthalmia or anophthalmia, micrognathia, and pre-auricular tags are common clinical findings among the patients. Patent ductus arteriosus, Ventricular septal defect, Atrial septal defect, Dextrocardia, and Tetralogy of Fallot are all frequent cardiac defects. Midline anomalies of the central nervous system are also prevalent, with alobar holoprosencephaly being the most common defect. Postaxial polydactyly, congenital talipes equinovarus, and rocker-bottom feet are all common extremities abnormalities. The lungs, liver, kidneys, genitourinary tract, digestive tract, and pancreas are among the other organ systems impacted by the disorder. Cryptorchidism, hypospadias, labia minora hypoplasia, and a bicornuate uterus are defects in these organ systems that affect more than half of Patau syndrome individuals. Omphalocele, incomplete colon rotation, Meckel diverticulum, polycystic kidney, hydronephrosis, and horseshoe kidney are abnormalities in these organ systems that affect less than half of Patau syndrome patients. Patients who survive childhood suffer significant psychomotor problems, inability to flourish, intellectual disabilities, and seizures [5].

Due of the patients' uniformly poor prognosis despite treatment, intensive treatment for Patau syndrome is controversial.Due to facial deformities, infants diagnosed with Patau syndrome may require post-delivery oxygenation and ventilation, which may necessitate intubation or tracheostomy. Patients with common cardiac problems may require cardiac surgery to correct them. Other surgeries, like as herniorrhaphy, cleft lip surgery, feeding tube installation, or corrective orthopedic surgeries, may be necessary for common problems. Specialized nutritional feeds, seizure prevention, prophylactic antibiotics for urinary tract infections, and the usage of hearing aids are all possible treatments [3].

The standard of healthcare has mostly centered on family education, pain management, and the avoidance of invasive interventions. When it comes to selecting which, if any, treatments are acceptable, the combination of a high mortality rate and severe cognitive and emotional defects is a difficulty for healthcare professionals and family members. With earlier detection and better therapies, certain kids with trisomy 13 have a better chance of surviving into adolescence, according to recent studies; nonetheless, these children still face substantial mental challenges. This work has been reported in line with the SCARE 2020 Guidelines [6].

## 2 Case report

A two-day-old infant presented to the Outdoor Patient department with a present complaint of vomiting after every feed and respiratory difficulty since birth. The patient was delivered through normal spontaneous vaginal delivery at 40 weeks of pregnancy. He was the third child born to a healthy 26-year-old mother para-3 in non-consanguineous marriage. However, the mother had two miscarriages before, one in the first month of pregnancy and one on the 20th day of pregnancy. The patient's sibling was born normal, but one child died later. Moreover, his family background was unremarkable. Additionally, the patient's birth weight was 2.8 kg (3rd-5th percentile), his length was 48 cm (10th-25th percentile), and his occipitofrontal circumference was 30 cm (3rd percentile). APGAR score was 10/10 at the time of delivery. He had no significant past medical or surgical history and did particularly not have a personal or family history of hypercoagulability or heart disorders.

The patient was thoroughly examined. The newborn examination revealed multiple dysmorphic characteristics: low set ears, cleft lip, cleft palate, right club foot, and rock bottom deformity. His vital signs, including pulse rate of 126 beats/minute, respiratory rate of 57/minute, and oxygen saturation (room air) of 96%, were noted, respectively. The chest was bilaterally clear. He was mentally active. His abdomen was soft and non-tender. No prominent abdominal distension was noted. However, pan systolic murmurs were heard on the left sternal border, and an apex beat with thrill was heard on the fifth intercostal space.

His complete blood count examination was done. All complete blood count findings were found to be abnormal except for differential leukocyte count and mean corpuscular hemoglobin concentration (32.7 g/dl). Hemoglobin levels were highly increased up to 19.2g/dl, and Total leukocyte count was found to be raised to 14600/cumm. However, the platelet count was found to be dramatically reduced to up to 74000/cumm. Similarly, HCT (Hematocrit), MCV (Mean corpuscular volume), Mean corpuscular hemoglobin was 58.7%,101 fl, and 33.3 pg, respectively.

Furthermore, all abdominal ultrasound findings were unremarkable except for the left kidney, which showed mild hydronephrosis. Considering the patient's age and the presence of a pan systolic murmur, echocardiography was performed. Echocardiography revealed situs solitus, levocardia, atrioventricular and ventriculoarterial concordance, atrial septal aneurysm with patent foramen ovale shunting left to right, large inlet Ventricular septal defect with bidirectional shunt, large PDA with bidirectional shunt, single right Superior vena cava with left innominate veins, severe pulmonary hypertension.

From the above-given laboratory reports and investigations, it was clear that the patient was suffering from Patau syndrome with congenital heart defects as echocardiography revealed clear findings. Moreover, visible dysmorphic features were observed on the patient body, such as the cleft palate, low set ears, cleft lip, and right club foot. This Echocardiography report and physical examination findings verified the ultimate diagnosis as Patau syndrome associated with congenital heart defects. On confirmation of diagnosis, the patient was transferred to the nursery ward. As a result of this diagnosis, the patient's first care strategy included oxygen administration to increase the oxygen saturation of the patient. Additionally, a nasogastric tube was passed, and the patient was given a feed every 5 seconds per 2 h. He was given IV Claforan 125mg twice a day, IV Ampicillin 125 mg two times a day, IV Lasix 5mg twice a day, and oral tablet Capril 1 cc, Tablet Aldactone 1 cc. The patient was also given 10 percent dextrose water 11ml/hr continuously. This medication administration was continued till the next day. On next day, IV Claforan, IV Ampicillin, and IV Lasix were given thrice a day. While tablet Capril, Tablet Aldactone, were given two times a day. The patient surgical plan of care included pulmonary binding, PDA ligation, VSD closure, and repair of ASA with disbanding of the pulmonary artery.

## 3 Discussion

In infants, trisomy 13, also known as Patau syndrome, is the third most prevalent autosomal trisomy. It is caused by the presence of an additional chromosome 13 as a result of translocation or nondisjunction of chromosomes. According to the studies, the incidence rate of Patau syndrome (Trisomy 13 syndrome) is 1/12.000–1/29.000 live births [7]. However, spontaneous abortions are more prevalent in mothers than live births [8]. Additionally, studies have reported that patients with Patau syndrome present with cleft lip and palate congenital heart defects, omphalocele, and holoprosencephaly. In our case, dysmorphic features such as the cleft palate and cleft lip were noted, and severe congenital heart defects were observed. Moreover, cardiac abnormalities, particularly patent ductus arteriosus, atrial septal defect, and ventricular septal defect being the most frequently encountered anomalies, have been reported in up to 80% of patients [9]. Our report provided similar findings. Furthermore, renal pathology, specifically cystic dysplasia, has been documented in more than 30% of patients [10]. However, in our case, only renal hydronephrosis was noted.

Studies have also reported that up to 50% of patients with Patau syndrome show significant ocular pathology, including cataracts, microphthalmia, and colobomas [11]. No such ocular deficit was noted in our study. Limb deficits such as limb deficiency, clinodactyly, and postaxial polydactyly, Italy, have also been reported in previous studies. Our patient was also diagnosed with clubfoot. Risk factors for Patau syndrome include a family history of Patau syndrome and Maternal age [12]. However, no such risk factor was observed in our case study as mother of the infant was only 26 years old.

The treatment options for Patau syndrome with congenital heart defects include oxygenation, the use of medications, repair of cardiac abnormalities, feeding tube placement, Herniorrhaphy, and cleft lip corrective orthopedic procedures [13]. Additionally, customized dietary feeds, prophylactic medicines for urinary tract infections, and the use of hearing aids may be undertaken. Despite rigorous management, median survival in the most recent cohorts of patients is only 733 days [13].

While it is well acknowledged that affected infants have a terrible prognosis and will have major disabilities if they survive, the current debate has raised questions about the level of care that should be provided to them. As children with Patau syndrome do not survive for a longer period, families and healthcare providers who promote life-saving procedures have been challenging traditional conceptions of palliative care. A consideration of this debate is beyond the scope of this report; however, it is suggested that when counseling families at the time of diagnosis and thereafter, a balanced approach be used, and that parental autonomy must be seriously addressed. Each situation should be handled on an individual basis, with individualized treatment based on the family's social and financial means, as well as the child's best interests. Genetic counseling is helpful not only in gaining a better knowledge of the diagnosis and its implications but also in assisting families in making difficult decisions about future care and family planning.

## 4 Conclusion

Patau syndrome is the third most frequently occurring trisomy in infants. A patient with Patau syndrome can present with clinical manifestations of cleft palate, cleft lip, limb impairments, and congenital heart defects. Advanced mother age and previous family history are major risk factors for Patau syndrome. Additionally, the current mainstay of treatment for Patau syndrome includes the supply of oxygen, medications, cardiac surgeries, and customized dietary feeds. Despite early diagnosis and management prognosis is poor for a patient suffering from Patau syndrome. However, genetic counseling might be advantageous in gaining a better understanding of the diagnosis and its consequences and also in helping families in making difficult choices about future care and family planning.

## Ethical approval

N/A.

## Sources of funding

N/A.

## Author contribution

All authors have contributed equally.

## Research registration (for case reports detailing a new surgical technique or new equipment/technology)

N/A.

## Guarantor

Ubaid khan is guarantor of this report.

## Consent

Written informed consent was obtained from the patient for publication of this case report and accompanying images. A copy of the written consent is available for review by the Editor-in-Chief of this journal on request.

## Provenance and peer review

Not commissioned, externally peer-reviewed.

## Declaration of competing interest

There is no conflict of interest.
